# Supplementary material for: Label Free Glucose Electrochemical Biosensor Based on Poly(3,4-ethylenedioxy thiophene):Polystyrene Sulfonate/Titanium Carbide/Graphene Quantum Dots
Source: Biosensors (Basel). 2021 Aug 7;11(8):267. doi: 10.3390/bios11080267 (PMC8393679; doi:10.3390/bios11080267)
Supplement: Supplementary file 1 [file biosensors-11-00267-s001.zip › biosensors-1220304-supplementary.pdf]

biosensors

Supporting Information

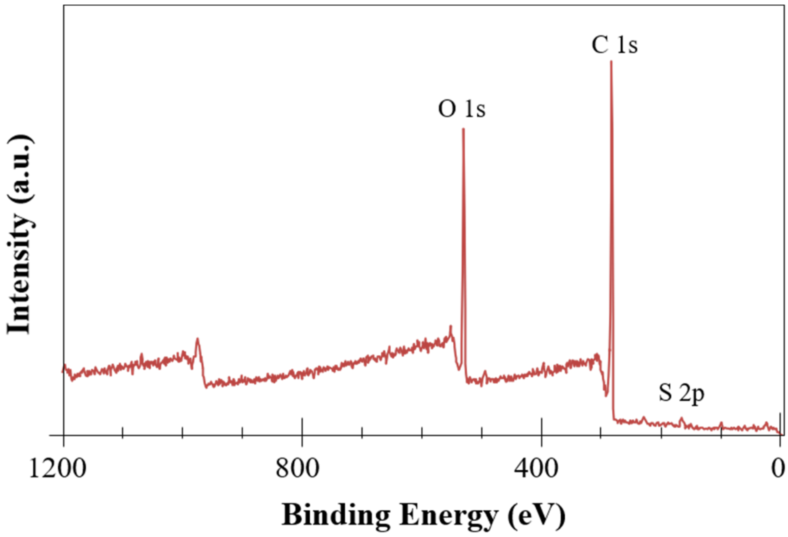

Figure S1. The general survey of the XPS spectrum of the PEDOT:PSS/Ti_3C_2/GQD with three strong binding energy peaks of S 2p, C 1s, and O 1s.

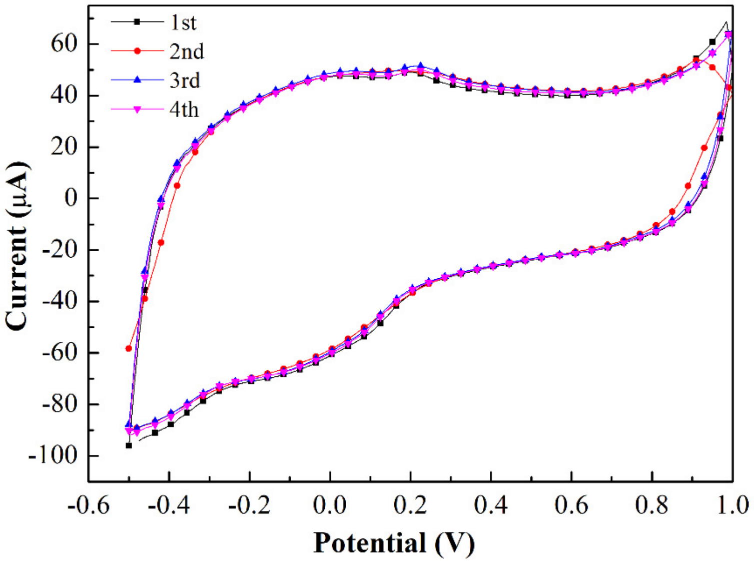

Figure S2. Repeatability of the modified electrode in four cycles after 1-month storage.
